# Initial Results of Peripheral-Blood Stem-Cell Mobilization, Collection, Cryopreservation, and Engraftment After Autologous Transplantation Confirm That the Capacity-Building Approach Offers Good Chances of Success in Critical Contexts: A Kurdish-Italian Cooperative Project at the Hiwa Cancer Hospital, Sulaymaniyah

**DOI:** 10.1200/JGO.17.00101

**Published:** 2017-12-15

**Authors:** Ignazio Majolino, Dereen Mohammed, Dastan Hassan, Francesco Ipsevich, Chra Abdullah, Rebar Mohammed, Angelo Palmas, Marco Possenti, Diana Noori, Dlir Ali, Harem Karem, Salah Salih, Michele Vacca, Claudia Del Fante, Angelo Ostuni, Andrea Frigato, Maria Speranza Massei, Annunziata Manna, Stefania Vasta, Marcela Gabriel, Marta Verna, Attilio Rovelli, Valentino Conter, Kosar Ali, Dosti Othman

**Affiliations:** **Ignazio Majolino**, **Francesco Ipsevich**, **Marco Possenti**, **Michele Vacca**, **Annunziata Manna**, **Stefania Vasta**, **Marta Verna**, **Attilio Rovelli**, and **Valentino Conter**, International Voluntary Service Association, Milan, Italy; **Ignazio Majolino**, **Francesco Ipsevich**, **Angelo Palmas**, **Marco Possenti**, **Michele Vacca**, **Claudia Del Fante**, **Angelo Ostuni**, **Andrea Frigato**, **Maria Speranza Massei**, **Annunziata Manna**, **Stefania Vasta**, **Marta Verna**, **Attilio Rovelli**, and **Valentino Conter**, Institute for University Cooperation, Rome, Italy; and **Dereen Mohammed**, **Dastan Hassan**, **Chra Abdullah**, **Rebar Mohammed**, **Diana Noori**, **Dlir Ali**, **Harem Karem**, **Salah Salih**, **Marcela Gabriel**, **Kosar Ali**, and **Dosti Othman**, Hiwa Cancer Hospital, Sulaymaniyah, Iraqi Kurdistan.

## Abstract

**Introduction:**

At Hiwa Cancer Hospital (Sulaymaniyah, Iraqi Kurdistan) after the center was started by a cooperative project in June 2016, autologous transplantation was developed.

**Patients and Methods:**

To develop the project, the capacity-building approach was adopted, with on-site training and coaching of personnel, educational meetings, lectures, on-the-job training, and the implementation of quality management planning.

**Results:**

Here, we report initial results of peripheral-blood stem-cell mobilization and collection of the first 27 patients (age 12 to 61 years; 19 males and 8 females; multiple myeloma, n = 10; plasma cell leukemia, n = 1; Hodgkin lymphoma, n = 12; non-Hodgkin lymphoma, n = 3; and acute myeloid leukemia, n = 1). Only three (11.5%) of 26 patients experienced a failure of mobilization. A median of 6.1 × 10^6^/kg CD34-positive cells per patient were collected (range, 2.4 to 20.8), with two apheretic runs. Twenty-four patients underwent autologous transplantation. All but one transplantation engrafted fully and steadily, with 0.5 and 1.0 × 10^9^/L polymorphonucleates on day 10.5 (range, 8 to 12) and day 11 (range, 9 to 15), respectively, and with 20 and 50 × 10^9^/L platelets on day 13 (range, 10 to 17) and day 17 (range, 2 to 44), respectively. More than 95% of patients are projected to survive 1 year after autograft.

**Conclusion:**

These data are the result of an Italian effort to establish in Iraqi Kurdistan a leading center for hemopoietic stem-cell transplantation. The capacity building approach was used, with on-site training and coaching as instruments for the development of provider ability and problem solving. With future limitations for immigration, this method will be helpful, especially in the field of high-technology medicine.

## INTRODUCTION

Hemopoietic stem-cell transplantation (HSCT) is effective for the treatment of many hematologic disorders.^1^ Unfortunately, not all countries have enough resources and expertise to establish an HSCT program.^2^ Iraqi Kurdistan recently entered a deep economic crisis that also involved the health system. We have previously described^3^ the capacity-building process that led to starting an HSCT center at Hiwa Cancer Hospital (HCH; Sulaymaniyah, Iraqi Kurdistan). Activity began in April 2016 and led to the first autologous transplantation in June and an allogeneic transplantation in October of the same year.

Here, we report an analysis of peripheral-blood stem-cell (PBSC) mobilization and collection of the first 27 patients and the engraftment times of 24 patients who underwent autologous transplantation. These results are comparable to those of major European Union and US centers, which confirms the value of capacity building as means to develop high-technology medical procedures in low-to-middle income countries.

## PATIENTS AND METHODS

### HSCT Center

This study was conducted at the recently established HSCT center of HCH, with six single-bed, HEPA-filtered, positive-pressure sterile rooms, four double-bed clean rooms, and an apheresis unit, with a Fresenius Comtec, an Amicus Fenwall cell separator (Fresenius, Kabi, Bad Homburg, Germany), and a manipulation laboratory for cell separation and cryopreservation.

### Capacity Building

The capacity-building approach is a conceptual approach^4^ that is focused on understanding and surmounting obstacles that prevent organizations from realizing sustainable development goals. This process was adopted at HCH, with on-site training and coaching of personnel for the duration of the project. In particular, in the first 2 months, educational meetings were organized for 55 health care professionals—physicians, nurses, biologists, and managers—with 60 lectures conducted. On-the-job training was developed, and quality management planning was implemented, with organizational charts, a documentation system, and verification of activities for continuous improvement. All procedures were written and coded, verified, and shared with local professionals. Indicators were set to periodically check the trends of the activities.

### Patients

Twenty-seven patients with multiple myeloma (MM), plasma-cell leukemia (PCL), Hodgkin lymphoma (HL), non-Hodgkin lymphoma (NHL), or acute myeloid leukemia (AML) were recruited to the program from June 2016 to March 2017 (Table 1). All patients received in-depth information on their disease and the HSCT procedure and provided written consent. The Ethical Committee of the College of Medicine, University of Sulaimani, approved the analysis and publication of the retrospective study data.

**Table 1:**
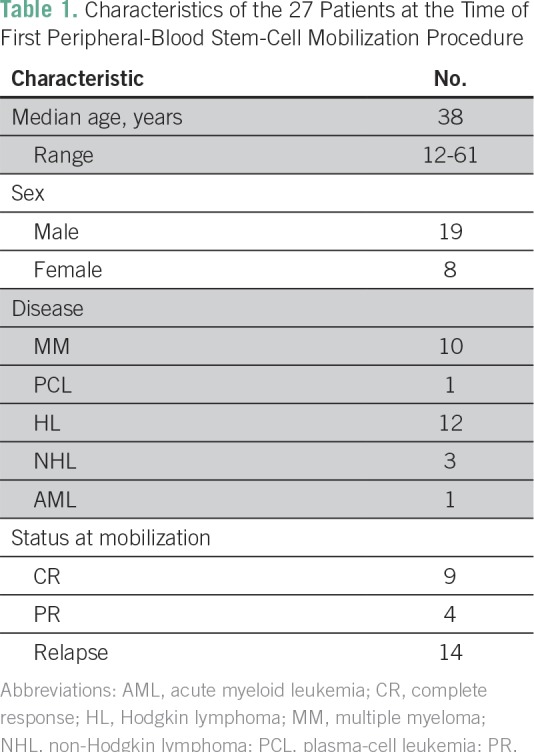
Characteristics of the 27 Patients at the Time of First Peripheral-Blood Stem-Cell Mobilization Procedure

### PBSC Mobilization

PBSC mobilization regimen was determined on the basis of disease and cell target. Initially, granulocyte colony-stimulating factor (G-CSF) alone 5 µg/kg twice a day^5^ (Sanofi, Paris, France) was administered to patients with MM, as the collection target was 5 × 10^6^/kg CD34-positive cells. Later, the target was set to 10 × 10^6^/kg CD34-positive cells to enable a double autograft, and intermediate (1.5 to 2 g/m^2^)^6^ or high-dose cyclophosphamide (4 g/m^2^) were used,^7^ always with G-CSF. Patients with lymphoma were mobilized mostly during their salvage chemotherapy. In HL, this was the BeGeV^8^ combination in eight patients and the IGeV^9^ in one patient. The mobilization/collection step followed the second or third course, and G-CSF 5 µg/kg twice a day was administered since day 5. A schedule of intermediate-dose cyclophosphamide plus G-CSF was also used in three patients. In patients with NHL, the rituximab plus dexamethasone, cisplatin, cytarabine salvage regimen was used,^10^ or intermediate-dose cyclophosphamide always followed by G-CSF. A single patient with AML was recruited for autograft. Fludarabine, cytarabine, and G-CSF^11^ was also used for mobilization. Details of each regimen are listed in Table 2. In all patients, G-CSF was continued until the collection target was reached.

**Table 2:**
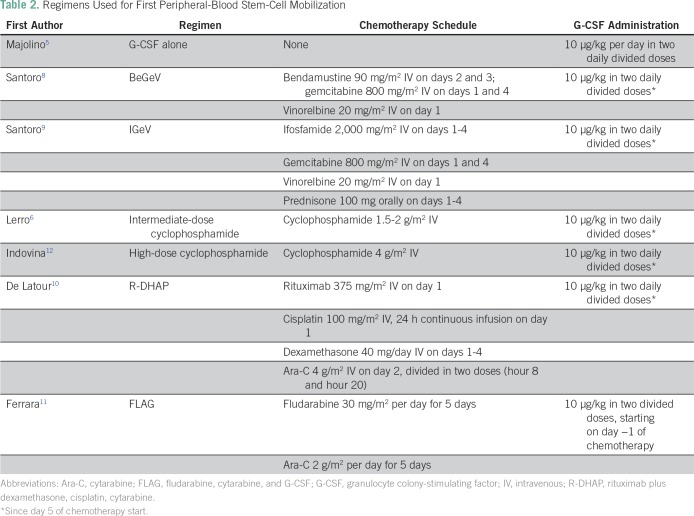
Regimens Used for First Peripheral-Blood Stem-Cell Mobilization

### CD34-Positive Cell Collection

After mobilization, blood cell counts were monitored. After chemotherapy-induced pancytopenia or, in the case of G-CSF alone, on day 4 since its beginning, CD34-positive cells were assessed daily by using a stem-cell enumeration kit (BD Biosciences, Brea, CA) and FACS Via flow cytometer (BD Biosciences). Initially, a double platform was employed,^13^ but a single platform was later used.^14^ Collections were usually started as CD34-positive cells rose > 20 × 10^6^/L, and only in a minority between 10 and 20 × 10^6^/L, using either the Fresenius Comtec or the Amicus Fenwall cell separator, two to three blood volumes per procedure. An algorithm was used for collection prediction.^15^ The target was 5 × 10^6^/kg body weight for each planned transplantation for collected CD34-positive cells.^16^ The number doubled for candidates of double autologous transplantation.

### PBSC Manipulation and Cryopreservation

A C-grade manipulation facility with a laminar flow hood was used. Cryopreservation was initially carried at −80°C in a mechanical freezer,^17^ but cells were later frozen in a liquid nitrogen tank in 10% DMSO (Sigma-Aldrich, St. Louis, MO) in autologous plasma using Fresenius Hemofreeze (Fresenius) bags. At the time of autograft, bags were rapidly thawed in a 37°C water bath and infused.

### Autologous Transplantation

For autologous transplantation, we used PBSC alone followed by high-dose chemotherapy. This schedule was based on disease and the availability of drugs. In patients with MM or PCL, patient received high-dose melphalan (140 mg/m^2^, n = 3; or 200 mg/m^2^, n = 7).^18^ In patients with HL or NHL, carmustine, etoposide, cytarabine, melphalan^19^ was the first choice for treatment (n = 7), but TEAM (n = 5)^20^ or cyclophosphamide, carmustine, and etoposide (n = 1)^21^ were used when carmustine was unavailable. TEAM was used in patients with AML. All patients received G-CSF 5 µg/kg since day +5 after autograft and until achievement of > 1.0 × 10^9^/L polymorphonucleates (PMNs).

### Transfusions

Blood products were irradiated (2.5 Gy) before transfusion. Packed RBCs were transfused when the Hb level was < 8 g/dL, while platelet concentrates, in the absence of bleeding or fever, were transfused when the platelet count was < 10.0 × 10^9^/L.

### Fever Management

At the onset of fever, blood cultures were obtained (one from the central venous catheter, if present) and the patient was immediately started on empirical antibiotics (piperacillin + tazobactam 400 mg/kg daily).

### Engraftment

Patients were monitored daily. Myeloid engraftment was defined as the attainment of ≥ 0.5 and 1.0 × 10^9^/L PMNs for 3 consecutive days. Platelet engraftment was defined as ≥ 20.0 and 50.0 × 10^9^/L platelets for 7 consecutive days and without support.

## RESULTS

### Capacity Building

A quality-based system was developed with a daily morning briefing; weekly seminars; and patient ward rounds, waiting list assessment, and periodical personnel re-evaluation by multiple-choice questionnaire.

### Mobilization and Collection

Results of the first PBSC mobilization and collection in 26 patients are summarized in Table 3. We considered failure to be CD34-positive cell peak < 10 × 10^6^/L, although in a patient with AML, we proceeded to apheresis despite a cell peak of 9.7 × 10^6^/L. In total, only three (11.5%) of 26 patients experienced a mobilization failure. Efficiency of the regimens was not significantly different. The day of the start of apheresis is reported in Table 3. Overall, with a median of two apheretic runs and 12,360 mL of blood (range, 3,575 to 17,100 mL) processed per run, 6.1 × 10^6^/kg CD34-positive cells per patient were collected (range, 2.4 to 20.8). The number of harvested CD34-positive cells was inferior to target only in three patients.

**Table 3:**
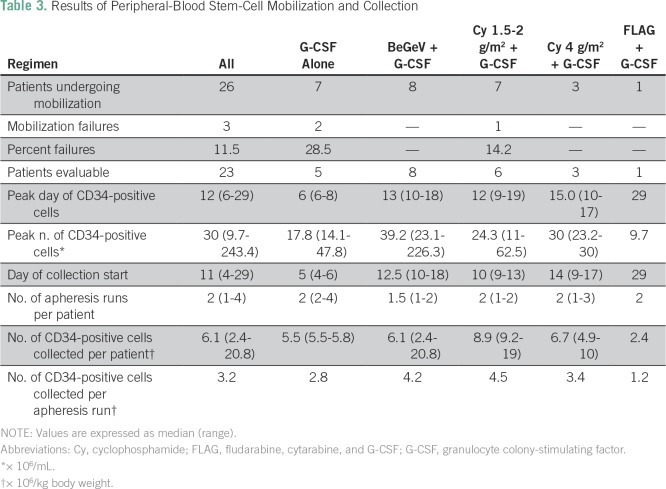
Results of Peripheral-Blood Stem-Cell Mobilization and Collection

### Second Mobilization Attempts

A second attempt at mobilization was made in two of the three patients who experienced failure, both of whom were patients with MM who experienced failure with G-CSF alone. One received cyclophosphamide 4 g/m^2^ plus G-CSF and collected 6.2 × 10^6^/kg CD34-positive cells, whereas the other was mobilized with cyclophosphamide 2 g/m^2^ plus G-CSF and collected 9.8 × 10^6^/kg CD34-positive cells.

### Autologous Transplantation and Engraftment

**Fig 1:**
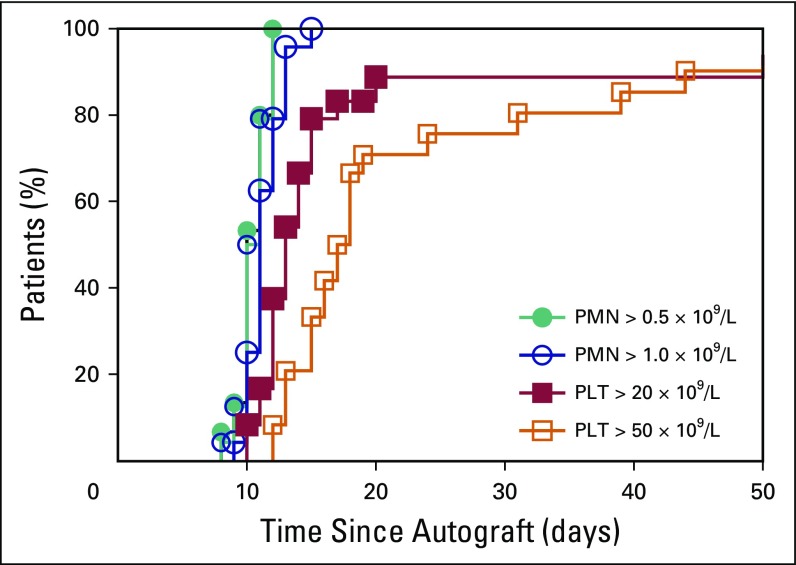
Engraftment as cell count achievement after autologous peripheral-blood stem-cell transplantation in 24 patients. Probability curves (Kaplan-Meier). PMN, polymorphonucleate; PLT, platelet.

**Fig 2:**
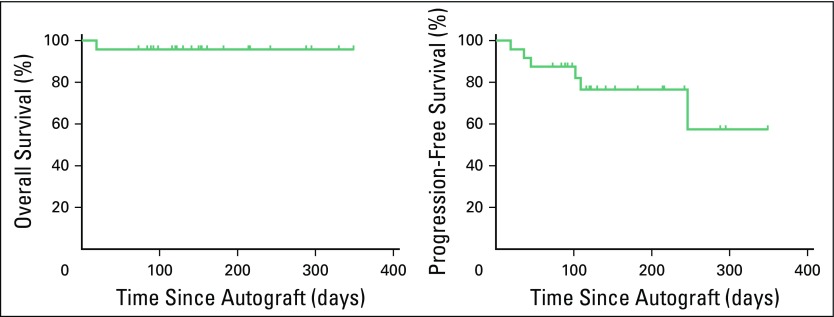
Overall survival (left) and progression-free survival (right) analysis of the entire population of patients who underwent autologous transplantation. Vertical ticks represent patients being observed.

Overall, 24 patients underwent autologous transplantation—nine patients with MM, one with PCL, 10 with HL, three with NHL, and one with AML. The majority (n = 16) of patients were in complete remission; they received 5.3 × 10^6^/kg CD34-positive cells (range, 4.6 to 20). All but one patient achieved full engraftment, with 0.5 and 1.0 × 10^9^/L PMN counts on day 10.5 (range, 8 to 12) and day 11 (range, 9 to 15), respectively, and with 20 and 50 × 10^9^/L platelets on day 13 (range, 10 to 17) and day 17 (range, 12 to 44), respectively. A probability curve for PMNs and platelet recovery is reported in Figure 1. Overall, patients experienced 2 days of fever > 38°C (range, 0 to 11) and received one packed RBC transfusion (range, 0 to 6) and two platelet concentrates (range, 0 to 11). A single 60-year-old female patient died early after the autograft (day +19) as a result of a dramatic cardiac failure, without full engraftment. This death was not clearly related to drug toxicity, as the high-dose regimen—carmustine, etoposide, cytarabine, melphalan—does not contain cyclophosphamide. All patients but one are alive at 150 days (range, 73 to 349) since autograft. As shown in Figure 2 (Kaplan Meier), > 90% of patients are projected to survive and almost 60% are free of progression at 1 year after transplantation.

## DISCUSSION

Use of autologous transplantation is effective in various hematologic neoplasms, such as MM, HL, NHL, and select cases of AML and solid tumors.^22^ PBSCs are now the standard; however, mobilization and collection of PBSCs represent critical steps.

**Fig 3:**
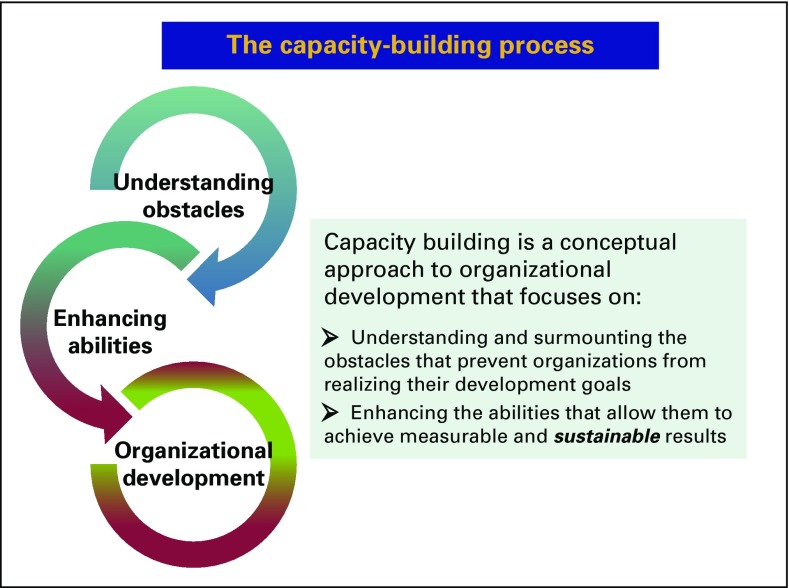
The capacity-building concept.

Here, we report the initial experience at HCH, the first oncology institution of Iraqi Kurdistan, where a capacity-building project was funded by the Italian Agency for Development Cooperation and approved by local health authorities. The predefined target patient population was a group of patients with thalassemia major, but autologous transplantation was assumed to be an intermediate step. In April 2016, an Italian team of experts steered a training program that covered all aspects of HSCT by means of lectures and coaching. The methodology was capacity building^4^ (Fig 3). In June 2016, the first autologous transplant was successfully performed, and the first allogeneic transplant in October 2016.

Mobilization of PBSCs in the blood was initially performed by G-CSF alone, but all protocols gave satisfactory results with a limited number (n = 3) of patients—always patients with MM—who experienced failure. Two of the three patients who experienced failure responded well to a different mobilization regimen. We confirmed that a new effective salvage combination for HL, named BeGeV^11^ can be successfully used as a PBSC mobilizing regimen. All eight patients who received BeGeV plus G-CSF demonstrated a high CD34-positive cell peak (median, 39.3) and collected the target cell number with a limited number of aphereses.

We assumed engraftment as an end point. Of the 24 patients who underwent autologous transplantation, only one did not achieve full engraftment as a result of sudden death on day +19. All other patients achieved full and steady early engraftment, with low transfusion support and limited days of fever. This reproduces the standard of the European Union and the United States, as confirmed by overall survival analysis (Fig 2), whereas the progression-free survival curve reflects patient referral, with transplants performed after repeated unsuccessful attempts. In future, with better transplant indications, results are expected to improve.

This study is the result of an Italian effort to establish a leading HSCT center in Iraqi Kurdistan. After the start of both the autologous and allogeneic transplantation programs, we now count seven patients with thalassemia and one patient with AML having undergone transplantation from HLA-identical siblings.^23^ We are planning a study to estimate the whole cost of mobilization, collection, cryopreservation, and autologous transplantation. These data are not available at the moment.

The capacity-building approach^4^ (Fig 3) is aimed at a sustainable development and strengthening of capacities through the enhancement of local skills. Our organization is based on on-site training, and, together with coaching, this represents an innovative and flexible method for sustainable activity in low-to-middle income countries as an alternative to the training performed abroad in specialized centers. With the current limitations for immigration, more on-site capacity-building projects will be developed.

With limited resources, it is essential to address the point of transplantation medicine. Despite the current economic crisis, the Kurdistan region is a territory rich in natural resources, with a universal health care system. In future, the situation could rapidly improve; however, as the government is spending more than 6 million USD/year to send patients abroad for HSCT, now seems to be the time to develop it locally.

We drove the training since the beginning. Before the clinical program was started, appropriate end points were assessed. In the present report, despite the limited number of patients and the short follow-up, we demonstrate that excellent results can be obtained even in difficult situations when a correct strategy, such as capacity building, is utilized form the start.
